# Dietary *Artemisia argyi* Inclusion Modulates Gastrointestinal Morphology, Immune-Related Indicators, and Bacterial Community Composition in Hu Sheep

**DOI:** 10.3390/ani16182904

**Published:** 2026-09-15

**Authors:** Beibei Chen, Huixiang Wang, Yiliyaer Abulajiang, Xiaoxue Chen, Yaqi Cui, Min Ren, Ruiping She, Xuna Ding, Haihong Jiao

**Affiliations:** 1 College of Animal Science and Technology, Tarim University, Alaer 843301, China; c15292302093@163.com (B.C.); worah0721@163.com (H.W.); 15739293934@163.com (Y.A.); chenxiaoxue0728@163.com (X.C.); 17509906679@163.com (Y.C.); xunading2023@126.com (X.D.); 2 Key Laboratory of Livestock and Forage Resources Utilization around Tarim, Ministry of Agriculture and Rural Areas, Alaer 843300, China; 3 Key Laboratory of Conservation and Utilization of Biological Resources in the Tarim Basin, School of Biological Sciences, Tarim University, Alaer 843301, China; renminlure@163.com; 4 Laboratory of Animal Pathology and Public Health, Key Laboratory of Zoonosis of Ministry of Agriculture, College of Veterinary Medicine, China Agricultural University, Beijing 100107, China; sheruiping@126.com

**Keywords:** *Artemisia argyi*, Hu Sheep, mucosal barrier, gut microbiota, immune function

## Abstract

Antibiotic-associated gastrointestinal dysbiosis can compromise microbial homeostasis and mucosal health, creating a need for natural feed additives that support gastrointestinal resilience. *Artemisia argyi* contains bioactive compounds with antimicrobial, antioxidant, and immunomodulatory properties, but its integrated effects on the gastrointestinal tract of ruminants remain insufficiently characterized. This study evaluated whether dietary *A. argyi* inclusion after a gentamicin-associated gastrointestinal disturbance modifies gastrointestinal morphology, serum immune-related indicators, intestinal inflammatory and barrier-related markers, and rumen and colonic bacterial communities in young Hu sheep. Forty one-month-old male Hu sheep were randomly allocated to four groups (*n* = 10 per group): control (Ctrl), gentamicin-challenge model (Model), low-inclusion *A. argyi* (ML, 1%), and high-inclusion *A. argyi* (MH, 3%). Following a 7-day modeling phase, during which Model, ML, and MH received gentamicin, and Ctrl did not, gentamicin administration was discontinued. Day 0 was defined as the baseline sampling point immediately after completion of the modeling phase. Blood was collected from all four groups on Day 0; immediately after this baseline collection, the 21-day dietary intervention began. During the intervention, Ctrl and Model received the basal diet, whereas ML and MH received the basal diet containing 1% and 3% *A. argyi*, respectively. Blood samples were subsequently collected from all sheep on intervention days 7, 14, and 21; therefore, serum analyses used *n* = 10 animals per group and repeated measurements from the same animals. Terminal tissue- and content-based laboratory analyses used *n* = 3 euthanized sheep per group. Dietary *A. argyi* inclusion was associated with improvements in selected gastrointestinal morphological indices, although responses differed by segment and inclusion level. Serum immunoglobulins and cytokines showed time- and treatment-associated patterns rather than a uniform response. Intestinal qPCR responses were gene- and segment-specific, and immunohistochemical effects differed according to marker and quantitative metric. For microbiota, selected taxa differed among groups, whereas the genus-level PCoA analyses presented in the manuscript did not detect significant global separation in either rumen (R = 0.0803, *p* = 0.292) or colonic (R = −0.0185, *p* = 0.528) communities. Overall, dietary *A. argyi* inclusion was associated with selected host and microbial responses after gentamicin exposure, but the effects were not uniformly dose-dependent and did not establish generalized gastrointestinal recovery or microbial functional restoration. Larger analytical sample sizes and direct functional measurements are required for confirmation.

## Simple Summary

The health of the gastrointestinal tract is important for growth and productivity in sheep. Antibiotic exposure can disrupt gastrointestinal microbial homeostasis and alter mucosal structure and host responses. This study evaluated whether dietary inclusion of *Artemisia argyi* could mitigate gentamicin-associated gastrointestinal alterations in young Hu sheep. Forty one-month-old male Hu sheep were allocated to four treatment groups (*n* = 10 per group). After a 7-day gentamicin modeling phase in the Model, ML, and MH groups, gentamicin was discontinued, and the 21-day dietary intervention began (day 0). The ML and MH diets contained 1% and 3% *A. argyi*, respectively, whereas the Ctrl and Model groups received the basal diet. Blood samples were collected from all enrolled sheep on days 0, 7, 14, and 21, providing *n* = 10 animals per group for longitudinal serum analyses. Terminal laboratory outcomes based on euthanized animals used *n* = 3 biological replicates per group. *A. argyi* inclusion was associated with segment- and endpoint-specific morphological and immune-related responses and with differences in selected rumen and colonic bacterial taxa; however, global genus-level community separation was not significant in the PCoA analyses. These findings support further evaluation of *A. argyi* as a natural feed additive while emphasizing the limited terminal analytical replication and the need for functional validation.

## 1. Introduction

The gastrointestinal tract is central to nutrient digestion and absorption, immune defense, and host–microbial interactions in ruminants [1,2]. Young sheep are particularly susceptible to gastrointestinal disturbances because rumen development, microbial colonization, epithelial-barrier maturation, and mucosal immune development are still progressing during early life [2,3,4]. During this stage, dietary transition, environmental stress, pathogen exposure, and antimicrobial treatment can destabilize gastrointestinal homeostasis. Such disturbances may alter microbial community structure, impair mucosal morphology and epithelial integrity, and modify inflammatory and immune responses, ultimately affecting animal health and production performance [3,5].

Antimicrobial exposure is especially relevant to gastrointestinal dysbiosis because it can reduce susceptible commensal populations, disrupt ecological competition and microbial metabolite networks, and favor expansion of resistant or opportunistic taxa. These shifts can disturb host–microbe interactions and contribute to epithelial injury and inflammatory activation. Gentamicin was selected as the antibiotic challenge because experimental studies have shown that gentamicin exposure can perturb intestinal microbial diversity and community composition in animal models [6]. In the present study, gentamicin was administered only during the 7-day modeling phase before the dietary intervention. Model establishment was assessed clinically at the end of the challenge: Ctrl sheep maintained normal appetite and pellet-shaped feces, whereas Model sheep showed varying degrees of diarrhea, soft or watery feces, and depressed general condition. These observations were used as clinical evidence of an antibiotic-associated gastrointestinal disturbance before dietary *A. argyi* inclusion.

*Artemisia argyi* is a traditional medicinal plant rich in flavonoids, polysaccharides, phenolic acids, and volatile oils, which have been reported to possess antioxidant, antimicrobial, anti-inflammatory, and immunomodulatory activities [7,8]. Previous studies have shown that dietary inclusion of *A. argyi* or its extracts can modify intestinal morphology, immune-related responses, and gut microbial composition in several animal species [9,10,11,12]. These observations suggest that *A. argyi* has potential as a natural feed additive [10].

However, evidence regarding *A. argyi* inclusion in ruminants remains limited. In particular, few studies have simultaneously evaluated gastrointestinal morphology, epithelial barrier-associated indicators, immune-related responses, and bacterial communities after antibiotic-associated gastrointestinal disturbance [13]. Consequently, the integrated gastrointestinal response to dietary *A. argyi* inclusion in young sheep has not been comprehensively characterized.

Hu sheep are an important indigenous breed in China and are widely used in intensive production systems [14]. Because gastrointestinal development and microbial stabilization are still ongoing in young animals, nutritional strategies that support gastrointestinal resilience are of practical interest [15]. Accordingly, this study established a gentamicin-associated gastrointestinal disturbance model in young Hu sheep and evaluated whether dietary *A. argyi* inclusion affects gastrointestinal morphology, serum immune-related indicators, intestinal inflammatory and barrier-associated gene/protein markers, and rumen and colonic bacterial community composition. We hypothesized that dietary *A. argyi* inclusion after gentamicin exposure would alleviate selected gastrointestinal alterations and modify measured host and bacterial indicators relative to the Model condition.

## 2. Materials and Methods

### 2.1. Ethical Statement and Experimental Site

All animal experiments were conducted in accordance with the guidelines for the ethical treatment of experimental animals and were approved by the Animal Care and Use Committee of Tarim University. The experiment was conducted at a commercial sheep farm located in Xinjiang, China.

### 2.2. Animals, Experimental Design, and Dietary Treatments

After a 7-day adaptation period, the Ctrl group received no gentamicin, whereas the Model, ML, and MH groups received gentamicin sulfate by oral gavage for 7 consecutive days. Gentamicin administration ended before the dietary intervention. Day 0 was the baseline sampling point immediately after the modeling phase. Blood was collected from all four groups on Day 0; immediately after this collection, the 21-day dietary intervention began. From after the Day 0 baseline collection through Day 21, Ctrl and Model were fed the basal diet, whereas ML and MH received the basal diet containing 1% and 3% *A. argyi*, respectively. Thus, the modeling and dietary-intervention phases were sequential and did not overlap.

After a 7-day adaptation period, the Ctrl group received no gentamicin, whereas the Model, ML, and MH groups received gentamicin sulfate injection by oral gavage to establish an antibiotic-associated gastrointestinal disturbance model. The gentamicin sulfate injection had a labeled concentration of 0.2 g/10 mL (20 mg/mL) and was administered at 0.5 mL/kg body weight per administration, equivalent to 10 mg/kg body weight per administration, twice daily (a total daily dose of 20 mg/kg body weight) for 7 consecutive days. Clinical condition, appetite, and fecal consistency were monitored during the modeling phase; at the end of the 7-day challenge, diarrhea together with soft or watery feces and depressed general condition in Model, in contrast to normal appetite and pellet-shaped feces in Ctrl, was used as clinical evidence of gastrointestinal disturbance. Gentamicin administration ended before the dietary intervention. Day 0 was defined as the first day after completion of the 7-day modeling phase. From day 0 to day 21, Ctrl and Model were fed the basal diet, whereas ML and MH received the basal diet containing 1% and 3% *A. argyi* pellets, respectively. Thus, the modeling and dietary-intervention phases were sequential and did not overlap.

All sheep were managed under the same farm conditions at Xinjiang Wushi County Zhenxingyuan Animal Husbandry Co., Ltd. (Shihezi, China), fed twice daily at 08:00 and 18:00, and allowed free access to feed and water. During adaptation and modeling, all sheep received the 5% fattening-sheep compound premix produced by Xinjiang Taikun Feed Co., Ltd. (Changji, China). During the formal 21-day dietary intervention, the basal diet was a commercial complete feed formulated for early-fattening meat sheep (product 198; Xinjiang Taikun Feed Co., Ltd., China). The product label and manufacturer inspection report available for this study provided guaranteed nutrient specifications but did not provide the ingredient list or ingredient-level proportions. Consequently, exact ingredient proportions could not be reported and were not estimated. The available manufacturer-declared nutrient specifications are presented in Table 1.

### 2.3. Preparation of the A. argyi Feed Additive

*Artemisia argyi* material was obtained from the Tarim University experimental station and collected in June 2024. After removal of impurities, the material was dried, ground, passed through a 40-mesh sieve, processed into *A. argyi* pellets using a feed granulator, and stored sealed in a cool, dry place until dietary inclusion.

### 2.4. Sample Collection

For terminal laboratory endpoints, three biological samples per treatment group were analyzed (*n* = 3/group). At the end of the 21-day dietary intervention, three sheep from each treatment group (12 animals in total) were euthanized for terminal sampling; the remaining enrolled animals were not used for the terminal tissue and gastrointestinal-content analyses reported here. Tissues and contents from the three euthanized sheep per group were used for H&E histomorphometry, SEM, qPCR, ileal immunohistochemistry, and 16S rRNA sequencing. For H&E morphometry, five well-oriented fields from each section were averaged to obtain one sheep-level value and were not treated as independent biological replicates. Before terminal sampling, the selected sheep were fasted for 24 h, and water was withheld for 2 h. They were then euthanized by carotid exsanguination, after which gastrointestinal tissues and contents were collected immediately.

Immediately after euthanasia, rumen, duodenum, jejunum, and ileum tissues were collected from each of the three terminally sampled sheep per group for histological analysis. Rumen and duodenal tissues were additionally collected for SEM. Duodenal, jejunal, and ileal tissues were collected for qPCR, whereas ileal tissue was used for immunohistochemistry. Histological and IHC samples were fixed in 10% neutral buffered formalin; SEM specimens were fixed in 2.5% glutaraldehyde; and qPCR tissues were rinsed with PBS, snap-frozen in liquid nitrogen, and stored at −80 °C until analysis.

Blood was collected from all enrolled sheep on days 0, 7, 14, and 21 of the dietary-intervention period. Thus, serum ELISA analyses used *n* = 10 animals per treatment group, with the same animals sampled repeatedly at each time point. Jugular venous blood was collected into tubes without anticoagulant, allowed to stand for 30 min at room temperature, and centrifuged at 3000 r/min for 10 min; serum was stored at −20 °C until ELISA. Serum immunoglobulins and cytokines were analyzed as repeated-measures outcomes.

For microbiota analysis, rumen ventral-sac contents and colonic contents were collected on day 21 from the same three terminally sampled sheep per treatment group. Rumen contents were collected aseptically, thoroughly mixed, and filtered through four layers of sterile gauze to obtain liquid and solid fractions. Colonic contents were collected aseptically with a sterile spatula. Samples were immediately frozen in liquid nitrogen and transferred to −80 °C for storage, with aliquoting used to minimize repeated freeze-thaw cycles.

### 2.5. Histological Analysis (H&E Staining)

Formalin-fixed rumen, duodenal, jejunal, and ileal tissues were trimmed to approximately 0.5 × 0.5 × 0.5 cm, refixed in 10% neutral buffered formalin for approximately 20 h, washed in running water for 8 h, and dehydrated sequentially in 70%, 80%, 90%, 95%, and absolute ethanol. Samples were cleared in an ethanol:xylene mixture followed by xylene, infiltrated with molten paraffin, embedded with the tissue cross-section appropriately oriented, and sectioned at 5 μm. Sections were deparaffinized, rehydrated, stained with hematoxylin and eosin (H&E), dehydrated, cleared, and mounted. This paraffin-section/H&E workflow and section thickness are consistent with published intestinal histomorphometry methods used in Hu sheep and Hu lambs [16,17].

Slides were digitized using an SQS-40P slide-scanning imaging system (Shengqiang Technology, Shenzhen, China), and morphometric measurements were performed with the Im-ageViewer pathology viewing system. Small-intestinal measurements comprised villus height, crypt depth, and the villus height-to-crypt depth ratio (V/C); rumen measurements comprised papilla length, papilla width, and mucosal thickness. Villus height was defined as the distance from the villus tip to the villus-crypt junction, whereas crypt depth was defined as the depth of the invagination between adjacent villi. For each tissue section, five well-oriented, structurally intact fields without obvious processing damage were selected for measurement, and the measurements were averaged to obtain one animal-level value for each variable. Accordingly, the fields within a section were treated as subsamples rather than independent biological replicates; the sheep was the biological replicate, with *n* = 3 sheep per treatment group for the reported histomorphometric analyses.

### 2.6. Scanning Electron Microscopy (SEM)

Rumen and duodenal samples (approximately 1–2 mm^3^) were gently rinsed with PBS and fixed in 2.5% glutaraldehyde at 4 °C for 24 h. Samples were washed three times in 0.1 mol/L PBS (pH 7.4; 15 min each), dehydrated sequentially in 50%, 70%, 80%, 90%, 95%, and 100% ethanol for approximately 15–20 min per step, subjected to critical-point drying, mounted on specimen stubs, sputter-coated with gold, and examined by scanning electron microscopy. This preparation is consistent with established SEM assessment of ovine ruminal mucosa [18]. Images were used for qualitative assessment of rumen papillae and duodenal mucosal surface ultrastructure rather than as independent quantitative replicates. The available study records do not specify the SEM make/model; therefore, no unverified instrument identifier was assigned.

### 2.7. Serum Biochemical and Immune Indices

Serum IgA, IgG, IgM, IL-6, IL-10, and TNF-α concentrations were measured using commercial sheep ELISA kits according to the manufacturers’ instructions. Absorbance was read at 450 nm with an HBS-109A microplate reader (Nanjing Detie Experimental Equipment Co., Ltd., Nanjing, China), and concentrations were calculated from standard curves. Jiangsu Jingmei Biological Technology Co., Ltd. (Yancheng, China) was the documented supplier of the IgA, IgG, IL-6, and IL-10 kits. The available reagent inventory does not unambiguously preserve the supplier/catalogue identifiers for the IgM and TNF-α kits, and these details were not inferred from the other assays.

### 2.8. Quantitative Real-Time PCR (qPCR)

On day 21, duodenal, jejunal, and ileal tissues were collected for qPCR. Total RNA was extracted using an RNAeasy™ Animal RNA Extraction Kit (centrifugal-column format; Beyotime Biotechnology, Shanghai, China), and cDNA was synthesized using HiScript IV All-in-One UltraRT SuperMix for qPCR (Vazyme, Nanjing, China). Quantitative PCR was performed with Taq Pro Universal SYBR qPCR Master Mix (Vazyme) on an FQD-96C real-time fluorescence quantitative PCR instrument (Hangzhou Bioer Technology Co., Ltd., Hangzhou, China). Reverse transcription and amplification were performed according to the corresponding kit manufacturers’ instructions, consistent with qPCR-based assessment of intestinal barrier and inflammatory markers in Hu sheep [19]. The measured targets were IFN-γ, IL-6, IL-17, TNF-α, and Claudin-1, with β-actin used as the reference gene. Relative expression was calculated using the 2^−ΔΔCt^ method. Primer sequences that could be verified from the available study records are listed in Table 2.

### 2.9. Immunohistochemistry

Ileal tissue was fixed in 10% neutral formalin for 24 h, routinely dehydrated, cleared, paraffin-embedded, and sectioned at approximately 4 μm. After deparaffinization, rehydration, and antigen retrieval, sections were incubated overnight at 4 °C with rabbit primary antibodies against IgA (Affinity Biosciences (Changzhou, China)/Jiangsu Qinke Biological Research Center Co., Ltd. (Liyang, China), cat. no. DF8563), MUC2 (cat. no. BF3256), Occludin (cat. no. DF7504), and ZO-1 (cat. no. AF5145). All four primary antibodies were diluted 1:200. Bound primary antibodies were detected using an HRP-conjugated goat anti-rabbit IgG (H+L) secondary antibody from the same supplier (cat. no. S0001), which was also diluted 1:200, followed by chromogenic development, hematoxylin counterstaining, dehydration, and mounting. Positive immunoreactivity was quantified by image analysis as mean staining intensity and positive-area percentage (Area%). The observed staining in ovine ileal sections provides study-specific evidence of tissue labeling; manufacturer-validated species reactivity was not independently established in this study.

The immunohistochemical outcomes reported include mean staining intensity and positive-area percentage (Area%). The statistical analysis used the sheep as the biological replicate (*n* = 3 sheep per group).

### 2.10. Microbiota Analysis (16S rRNA Sequencing)

Rumen ventral-sac and colonic content samples collected on day 21 were submitted to Shanghai Majorbio Bio-Pharm Technology Co., Ltd. (Shanghai, China) for bacterial 16S rRNA gene sequencing. Microbial community genomic DNA was extracted using the E.Z.N.A.^®^ Soil DNA Kit (Omega Biotek, Norcross, GA, USA) according to the manufacturer’s instructions. DNA quality was assessed by 1% agarose gel electrophoresis, and DNA concentration and purity were determined using a NanoDrop 2000 spectrophotometer (Thermo Scientific, Waltham, MA, USA). The V3–V4 region of the bacterial 16S rRNA gene was amplified using barcoded primers 338F (5′-ACTCCTACGGGAGGCAGCAG-3′) and 806R (5′-GGACTACHVGGGTWTCTAAT-3′). Each 20 μL PCR mixture contained 4 μL of 5× FastPfu buffer, 2 μL of 2.5 mM dNTPs, 0.8 μL of each primer (5 μM), 0.4 μL of FastPfu DNA polymerase, approximately 10 ng of template DNA, and nuclease-free water. Amplification comprised initial denaturation at 95 °C for 3 min; 27 cycles of 95 °C for 30 s, 55 °C for 30 s, and 72 °C for 30 s; and a final extension at 72 °C for 10 min. Amplicons were examined by 2% agarose gel electrophoresis, purified, quantified using a QuantiFluor™-ST system, pooled in equimolar amounts, and paired-end sequenced on an Illumina NextSeq 2000 platform by Majorbio.

After demultiplexing, paired-end reads were quality-filtered using fastp v0.19.6 and merged using FLASH v1.2.11. Reads were trimmed when the mean quality score within a 50 bp sliding window fell below 20; reads shorter than 50 bp after quality control or containing ambiguous bases were removed. Paired reads were merged using a minimum overlap of 10 bp and a maximum mismatch ratio of 0.20 in the overlap region. Barcode mismatches were not permitted, and no more than two primer mismatches were allowed. High-quality sequences were denoised using the DADA2 plugin in QIIME2 v2020.2 to generate amplicon sequence variants (ASVs); sequences assigned to chloroplasts or mitochondria were removed. Taxonomic assignment was performed using a naive Bayes classifier against the SILVA 16S rRNA database v138. Alpha-diversity indices, including Chao1 richness, Shannon diversity, and Good’s coverage, were calculated using Mothur v1.30.1. Genus-level beta diversity was evaluated using Bray–Curtis dissimilarities and visualized by principal coordinate analysis (PCoA). Overall differences among treatment groups were evaluated by analysis of similarities (ANOSIM). Differentially abundant taxa were identified using linear discriminant analysis effect size (LEfSe), with an LDA score threshold >2.0 and *p* < 0.05. Bioinformatic and statistical analyses of the bacterial-community data were performed using the Majorbio Cloud platform (https://cloud.majorbio.com). No PICRUSt2 or other functional prediction was used for the conclusions reported in this study.

### 2.11. Statistical Analysis

Data processing and statistical analyses were performed using ImageJ 1.50 and Excel 2022 as applicable, followed by SPSS 27.0; GraphPad Prism 10 was used for graph preparation. Data are presented as mean ± SD. The experiment used a completely randomized treatment allocation. The individual sheep was the experimental unit for animal-level outcomes, and within-section fields were subsamples that were averaged before analysis. Treatment allocation comprised *n* = 10 sheep per group. Serum ELISA outcomes were obtained from all 10 sheep in each group on days 0, 7, 14, and 21, with the same animals repeatedly sampled over time. Terminal tissue/content outcomes were based on the three sheep euthanized per group on day 21 (*n* = 3/group). Data distribution and variance homogeneity were assessed before parametric analysis. Serum immunoglobulins and cytokines were analyzed as repeated-measures outcomes using repeated-measures ANOVA to evaluate group, time, and group × time effects, with one-way ANOVA at individual time points when required. Terminal single time point quantitative host outcomes were analyzed by one-way ANOVA followed by Duncan’s multiple-comparison procedure. Microbiome sequence processing, diversity analyses, ANOSIM, and LEfSe were performed as described in Section 2.10 and were not analyzed using the host-outcome ANOVA workflow. SEM images were interpreted qualitatively and were not treated as independent quantitative observations. *p* < 0.05 was considered statistically significant and *p* < 0.01 highly significant. No formal a priori power calculation was performed.

## 3. Results

### 3.1. Effects of Dietary A. argyi Inclusion on Gastrointestinal Morphology

**Figure 1:**
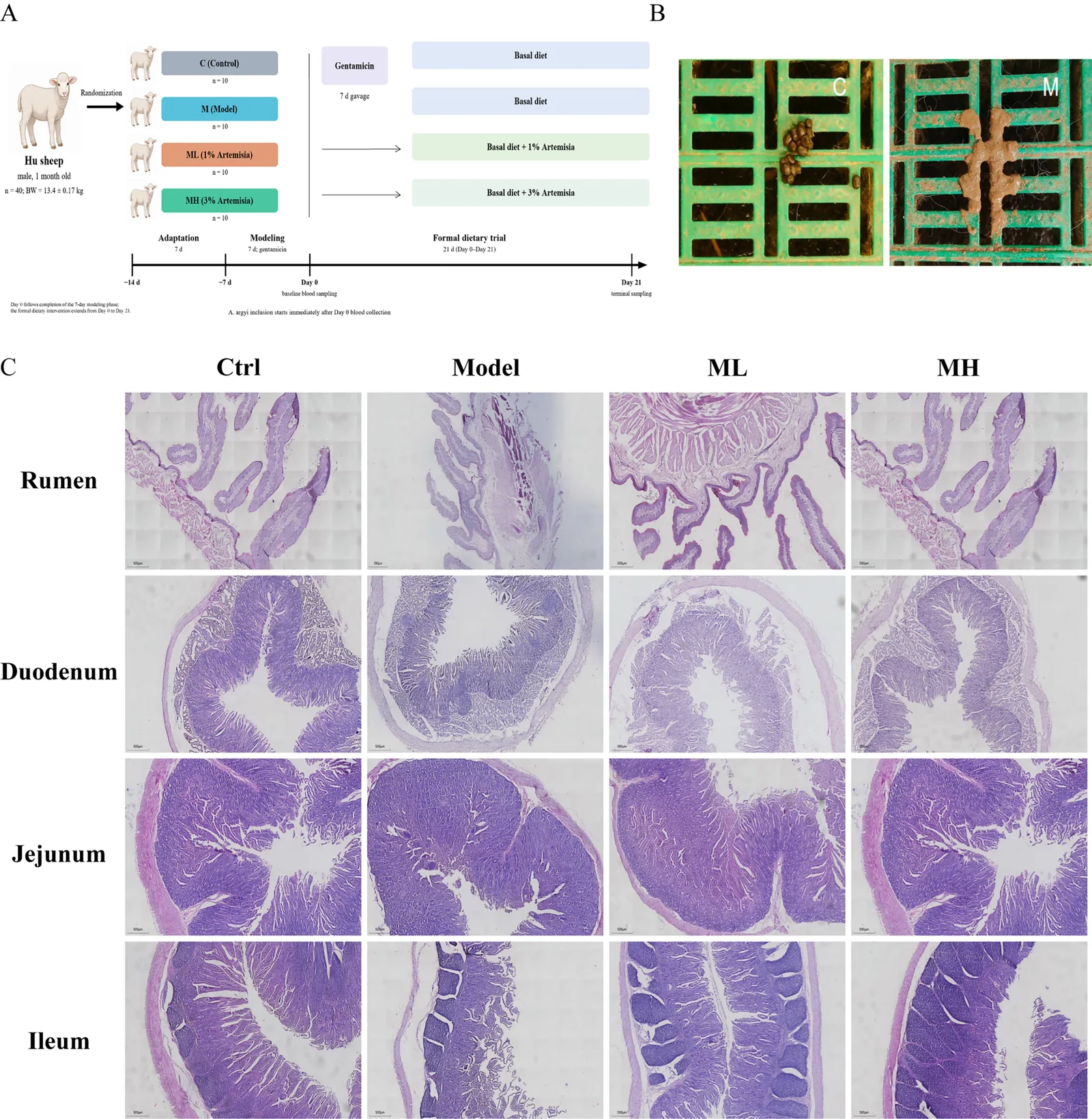
Experimental design and effects of dietary *Artemisia argyi* inclusion on gastrointestinal morphology in Hu sheep. (**A**) Experimental timeline and group allocation. Forty 1-month-old male Hu sheep were allocated to four groups (*n* = 10 per group). After adaptation, Model, ML, and MH received gentamicin for 7 consecutive days; Ctrl did not. Gentamicin was discontinued before Day 0. Day 0 denotes baseline blood sampling immediately after completion of modeling. The 21-day formal dietary intervention began immediately after the Day 0 blood collection; Ctrl and Model received the basal diet, whereas ML and MH received the basal diet containing 1% and 3% *A. argyi*, respectively. On day 21, three sheep per group were euthanized for terminal tissue and gastrointestinal content sampling, providing *n* = 3 biological replicates per group for terminal laboratory endpoints. (**B**) Representative fecal appearance after the 7-day modeling phase in Ctrl (C) and Model (M) sheep, illustrating the clinical model-establishment observations. (**C**) Representative H&E-stained sections of rumen, duodenum, jejunum, and ileum from Ctrl, Model, ML, and MH groups (40×). Images correspond to the analytical samples (*n* = 3 biological replicates per group); quantitative comparisons are reported in Table 3 and Table 4.

After the 7-day modeling phase, Ctrl sheep maintained normal appetite and pellet-shaped feces, whereas Model sheep showed varying degrees of diarrhea and soft or watery feces, consistent with the clinical model-establishment observations shown in Figure 1B. The experimental design and representative H&E images of the rumen, duodenum, jejunum, and ileum are shown in Figure 1. The Model group displayed qualitative gastrointestinal morphological alterations relative to Ctrl, including short-er/less organized rumen papillae and irregular intestinal villus morphology.

The ML and MH groups showed qualitative differences in several morphological features relative to Model; however, quantitative responses differed among endpoints and gastrointestinal segments and should be interpreted from Table 3 and Table 4 rather than from representative images alone.

The overall treatment effect on rumen papilla length was significant (*p* < 0.001; Table 3). According to the reported superscript letters, Ctrl (ab) and Model (a) share a letter and therefore are not significantly different in the post hoc comparison. ML (bc) and MH (c) do not share a letter with Model (a), whereas papilla width and mucosal thickness showed no significant overall treatment effects (*p* > 0.05).

Small-intestinal morphology showed significant overall treatment effects for duodenal villus length and crypt depth, jejunal villus length/crypt depth/V/C, and ileal villus length/crypt depth/V/C (Table 4). The response was not uniform across segments or inclusion levels.

In the duodenum, ML and MH had greater villus length than Model according to the reported superscripts, whereas crypt-depth differences did not show a consistent improvement pattern. In the jejunum, only ML had greater villus length than Model; MH and Model shared the same superscript. Both ML and MH had lower crypt depth than Model. For ileal villus length, Ctrl = a, Model = ab, ML = b, and MH = c; therefore, Model and ML share the letter b and are not significantly different, whereas MH does not share a letter with Model and differs significantly. The ileal V/C ratio was greater than Model only in ML, according to the reported superscripts.

For Table 3 and Table 4, groups sharing at least one superscript letter were interpreted as not significantly different, whereas groups with no shared superscript letter were interpreted as significantly different at *p* < 0.05.

### 3.2. Gastrointestinal Ultrastructure Observed by Scanning Electron Microscopy

**Figure 2:**
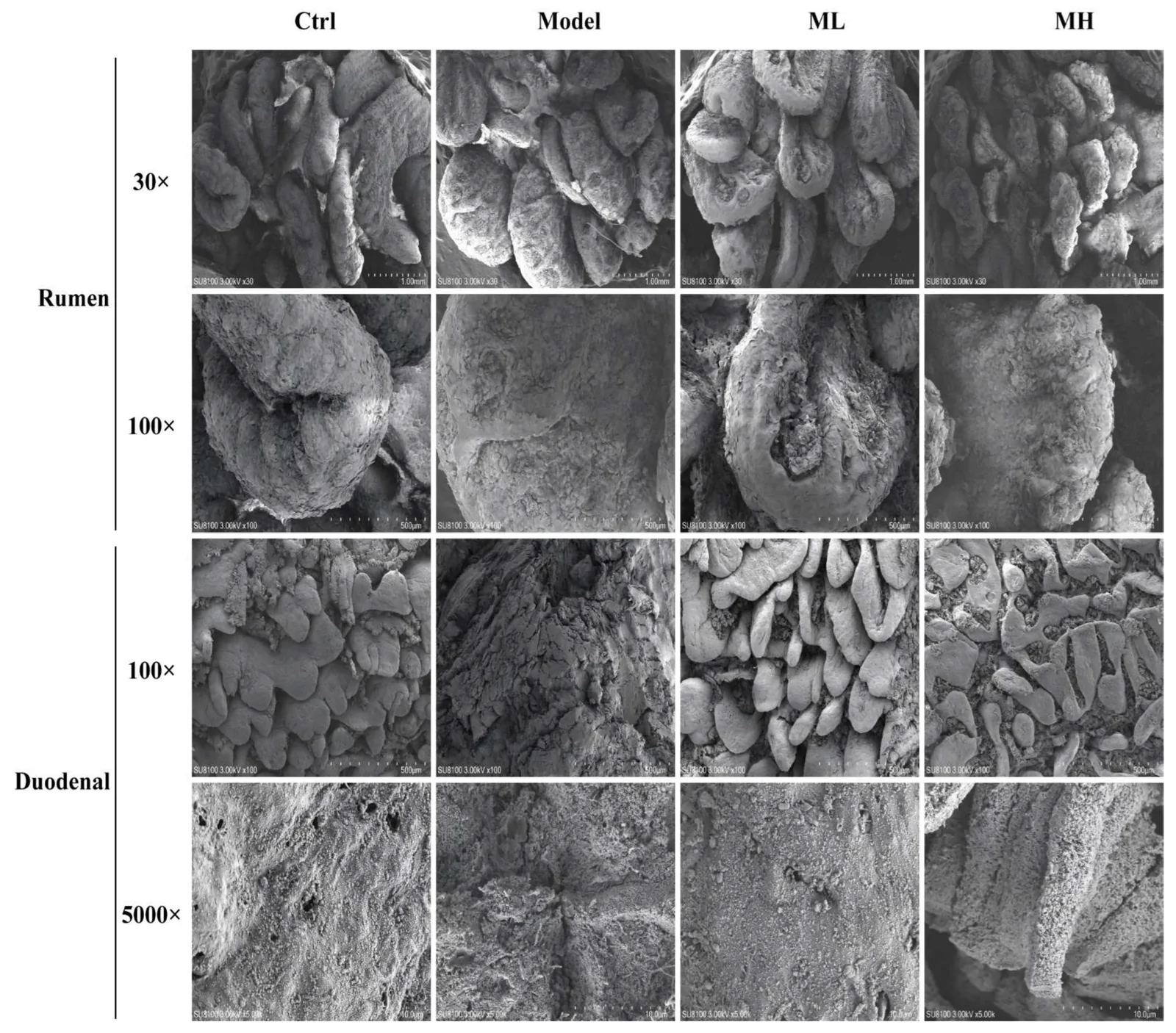
Effects of *Artemisia argyi* inclusion on gastrointestinal ultrastructure in Hu sheep. Representative SEM images show the surface morphology of rumen and duodenal mucosa among Ctrl, Model, ML, and MH groups. Rumen tissues were examined at 30× and 100× and duodenal tissues at 100× and 5000×. SEM was used for qualitative ultrastructural assessment; therefore, visual differences should be interpreted descriptively rather than as independent statistical effects. The reported analytical replication was *n* = 3 per group.

Representative SEM images are shown in Figure 2. Relative to Ctrl, the Model group showed a more irregular rumen mucosal surface and more pronounced epithelial shedding. In the duodenum, the Model group showed an uneven luminal surface, epithelial loss, exposed underlying tissue, and adherent exudative material. The *A. argyi* groups showed partial qualitative improvement in surface organization, although the response was not uniform between ML and MH. Because SEM was used qualitatively, these observations are descriptive rather than independent statistical evidence of treatment effects.

### 3.3. Effects of Dietary A. argyi Inclusion on Serum Cytokine Concentrations

**Figure 3:**
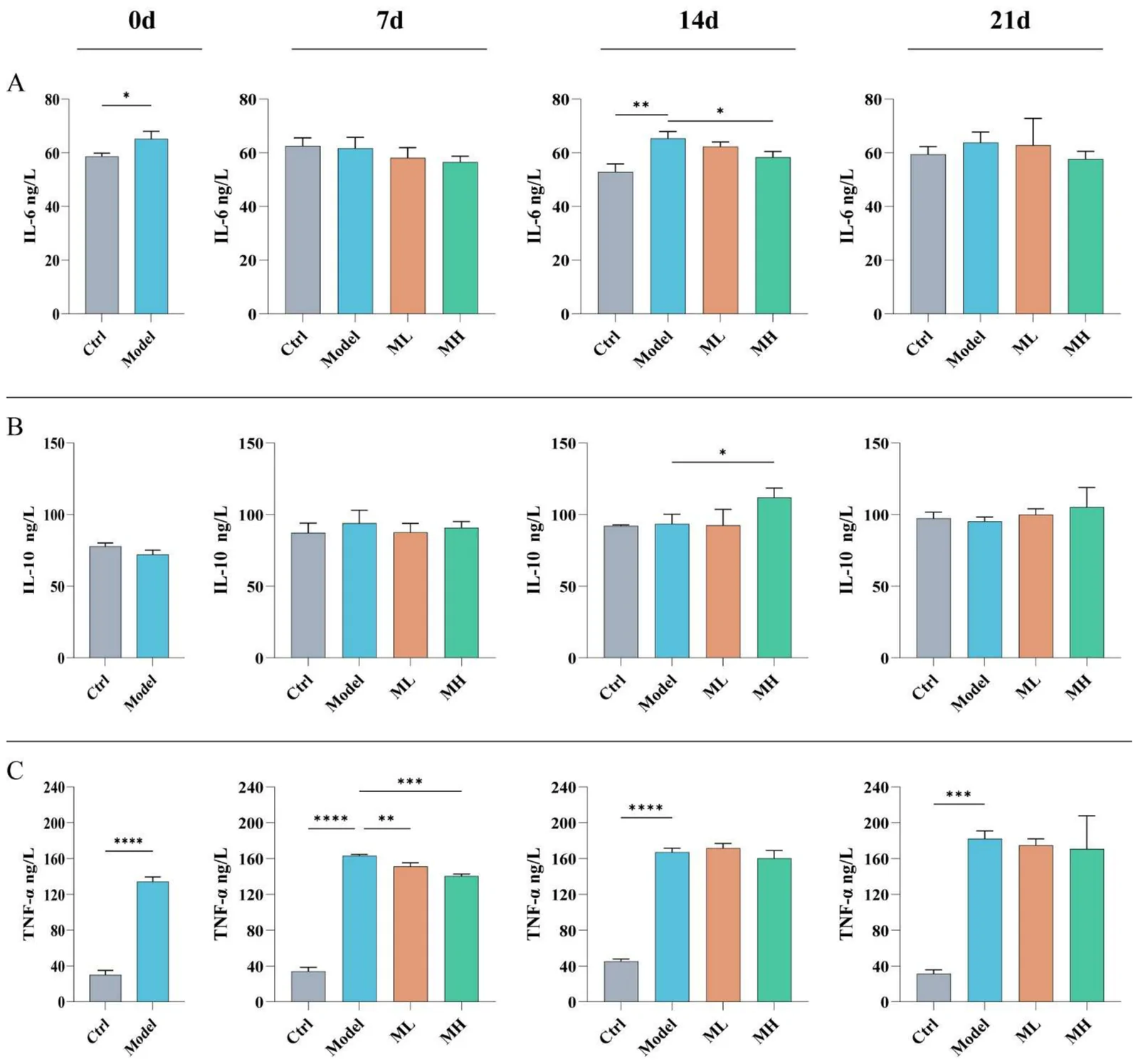
Effects of *Artemisia argyi* inclusion on serum inflammatory cytokine levels in Hu sheep. Serum cytokines were assessed at baseline (Day 0, immediately after modeling and before the dietary intervention) and on intervention days 7, 14, and 21. Blood was collected from all four groups on Day 0 and from all sheep thereafter; the same animals were sampled repeatedly. The Day 0 panels in Figure 3 display only Ctrl and Model to show the post-modeling baseline comparison before *A. argyi* exposure; ML and MH Day 0 serum samples were collected but were not plotted in these panels. All four treatment groups are displayed at intervention days 7, 14, and 21. Serum data represent *n* = 10 animals per displayed treatment group at each time point. (**A**) Interleukin-6 (IL-6) levels. (**B**) Interleukin-10 (IL-10) levels. (**C**) Tumor necrosis factor-alpha (TNF-α) levels. Statistical interpretation follows the repeated-measures analysis of group, time, and group × time effects, with time-point comparisons reported only when supported by the corresponding analysis. Asterisks indicate pairwise significance: * *p* < 0.05, ** *p* < 0.01, *** *p* < 0.001, and **** *p* < 0.0001.

Serum IL-6, IL-10, and TNF-α were evaluated over the dietary-intervention period (Figure 3). IL-6 changed relatively little and did not show a consistent treatment pattern. IL-10 generally increased as the trial progressed, with numerically higher late-stage values in MH. On day 0, TNF-α was markedly higher in Model than Ctrl (*p* < 0.0001); concentrations varied thereafter, and the MH value was numerically closer to Ctrl than Model on day 21. These data indicate time- and treatment-associated cytokine patterns rather than a uniform anti-inflammatory response across all markers and time points.

### 3.4. Effects of Dietary A. argyi Inclusion on Serum Immunoglobulin Concentrations

**Figure 4:**
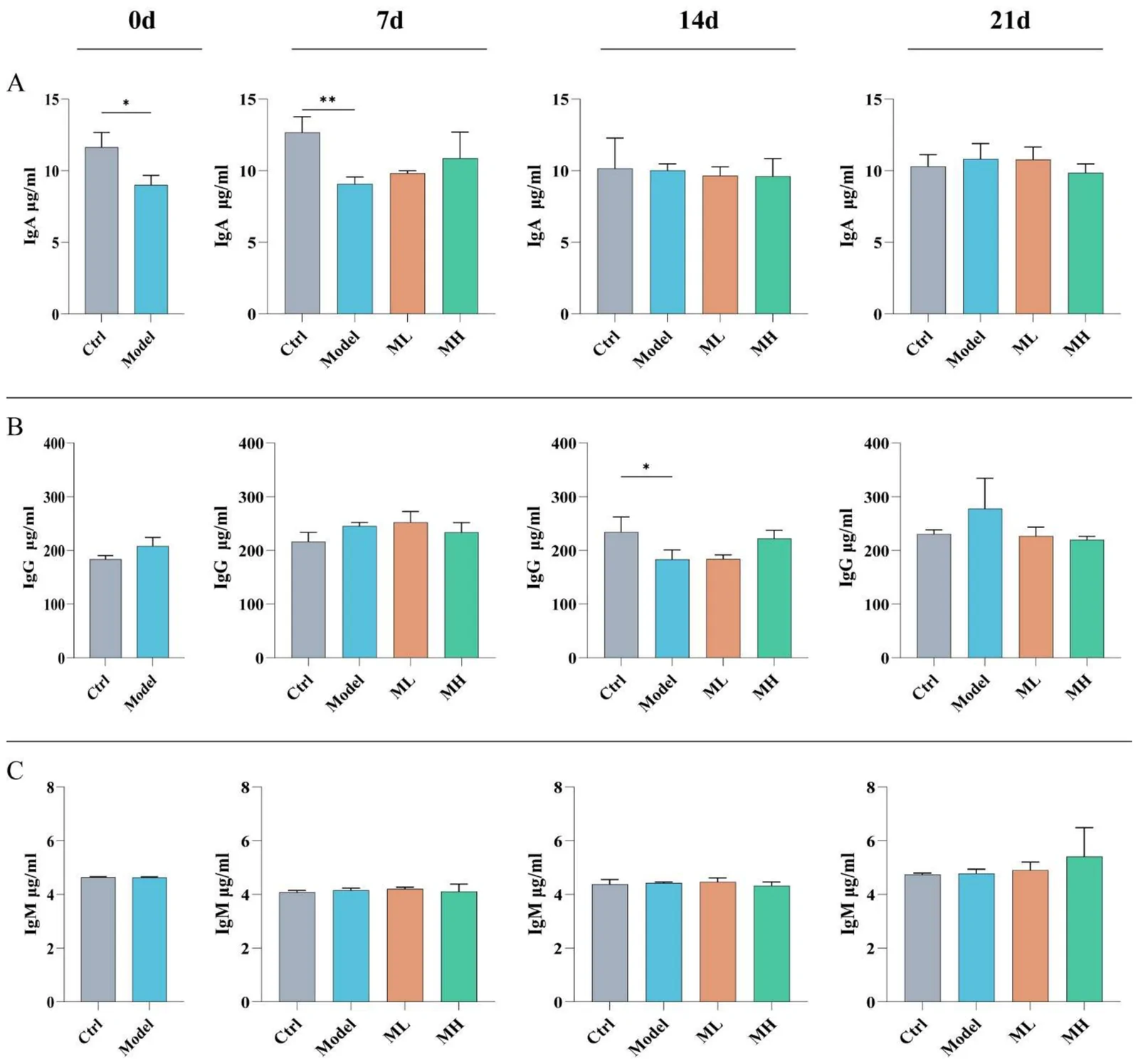
Effects of *Artemisia argyi* inclusion on serum immunoglobulin levels in Hu sheep. Serum immunoglobulins were assessed at baseline (Day 0, immediately after modeling and before the dietary intervention) and on intervention days 7, 14, and 21. Blood was collected from all four groups on Day 0 and from all sheep thereafter; the same animals were sampled repeatedly. The Day 0 panels in Figure 4 display only Ctrl and Model to show the post-modeling baseline comparison before *A. argyi* exposure; ML and MH Day 0 serum samples were collected but were not plotted in these panels. All four treatment groups are displayed at intervention days 7, 14, and 21. Serum data represent *n* = 10 animals per displayed treatment group at each time point. (**A**) Immunoglobulin A (IgA). (**B**) Immunoglobulin G (IgG). (**C**) Immunoglobulin M (IgM). The figure shows time- and treatment-associated serum immunoglobulin patterns. Interpretation is based on the repeated-measures/time-point analyses rather than visual trends alone. Data are presented as mean ± SD (*n* = 10 animals per group): * p < 0.05, ** *p* < 0.01.

Serum IgA, IgG, and IgM concentrations changed over the 21-day dietary-intervention period (Figure 4). IgG and IgM showed overall late-stage increases, with the numerical increase most evident in MH, whereas serum IgA varied more modestly and the groups tended to converge by day 21. Accordingly, the immunoglobulin data support time- and treatment-associated variation but should not be summarized as a uniform significant increase in all *A. argyi* groups. Pairwise statements at individual time points are limited to contrasts supported by the repeated-measures/time-point analyses.

### 3.5. Effects of Dietary A. argyi Inclusion on Intestinal Inflammatory- and Barrier-Related Gene Expression

**Figure 5:**
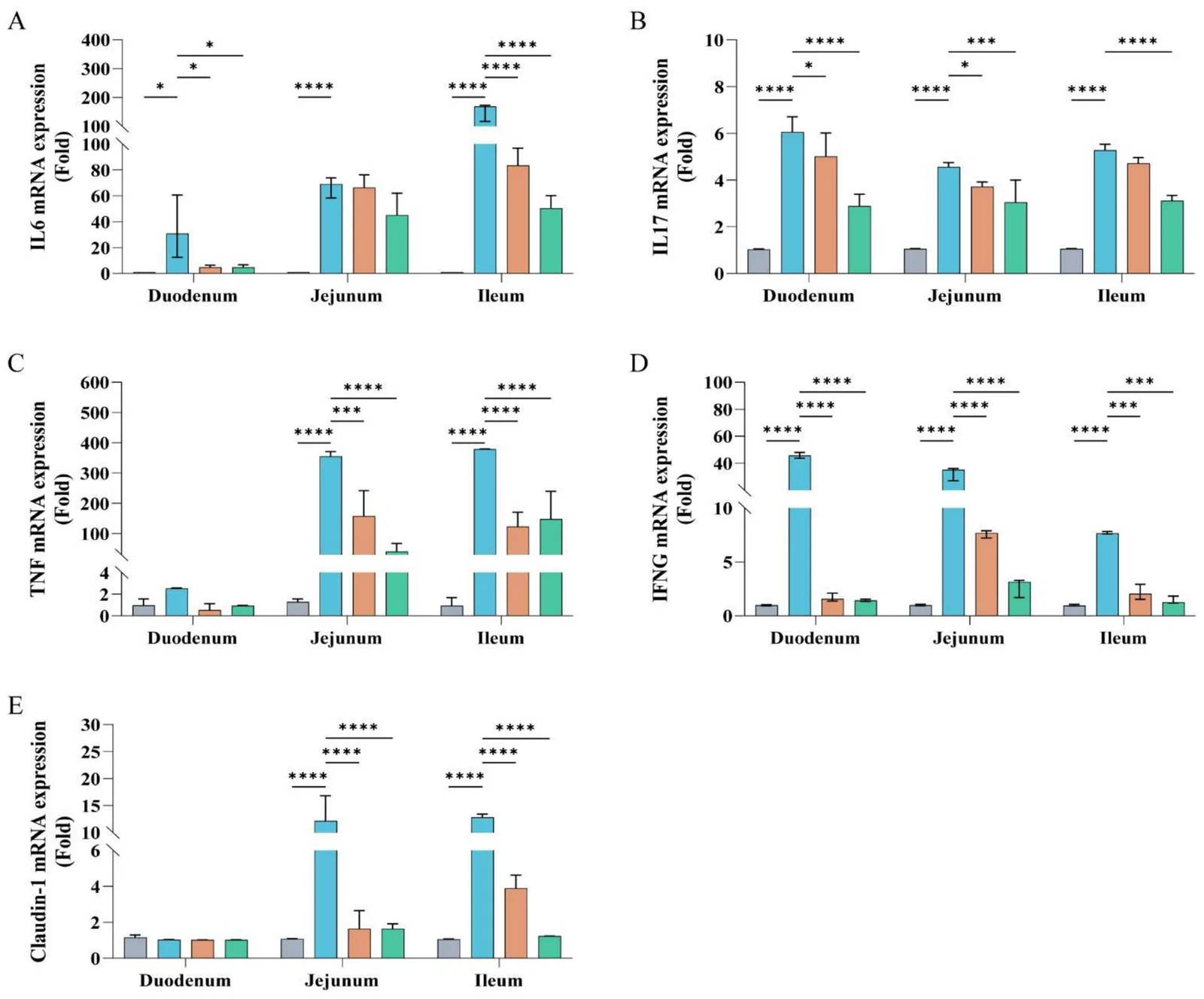
Effects of *Artemisia argyi* inclusion on inflammatory- and barrier-related gene expression in the duodenum, jejunum, and ileum of Hu sheep. Relative mRNA expression was determined by qPCR using β-actin as the reference gene and the 2^−ΔΔCt^ method. (**A**) IL-6 expression. (**B**) IL-17 expression. (**C**) TNF-α expression. (**D**) IFN-γ expression. (**E**) Claudin-1 expression. Data are presented as mean ± SD (*n* = 3 biological replicates per group): * *p* < 0.05, *** *p* < 0.001, **** *p* < 0.0001. Pairwise significance varies by gene and intestinal segment; significance brackets are shown in Figure 5.

Relative mRNA expression of IL-6, IL-17, TNF-α, IFN-γ, and Claudin-1 was evaluated in the duodenum, jejunum, and ileum by qPCR (Figure 5).

Across the three intestinal segments, the Model group generally showed elevated expression of several inflammatory genes relative to Ctrl, but the magnitude and pairwise significance varied by gene and segment. *A. argyi* inclusion reduced several of these model-associated elevations. The most consistent directional reductions were observed for IFN-γ, while IL-6, IL-17, and TNF-α responses varied among duodenum, jejunum, and ileum.

Claudin-1 expression showed little separation among groups in the duodenum but was elevated in the Model group in the jejunum and ileum and was lower after *A. argyi* inclusion. Thus, Claudin-1 should not be described as being increased by *A. argyi* in this experiment. Overall, the qPCR response was gene- and segment-specific rather than a uniform suppression or restoration across all targets.

### 3.6. Effects of Dietary A. argyi Inclusion on Ileal Immunohistochemical Markers

**Figure 6:**
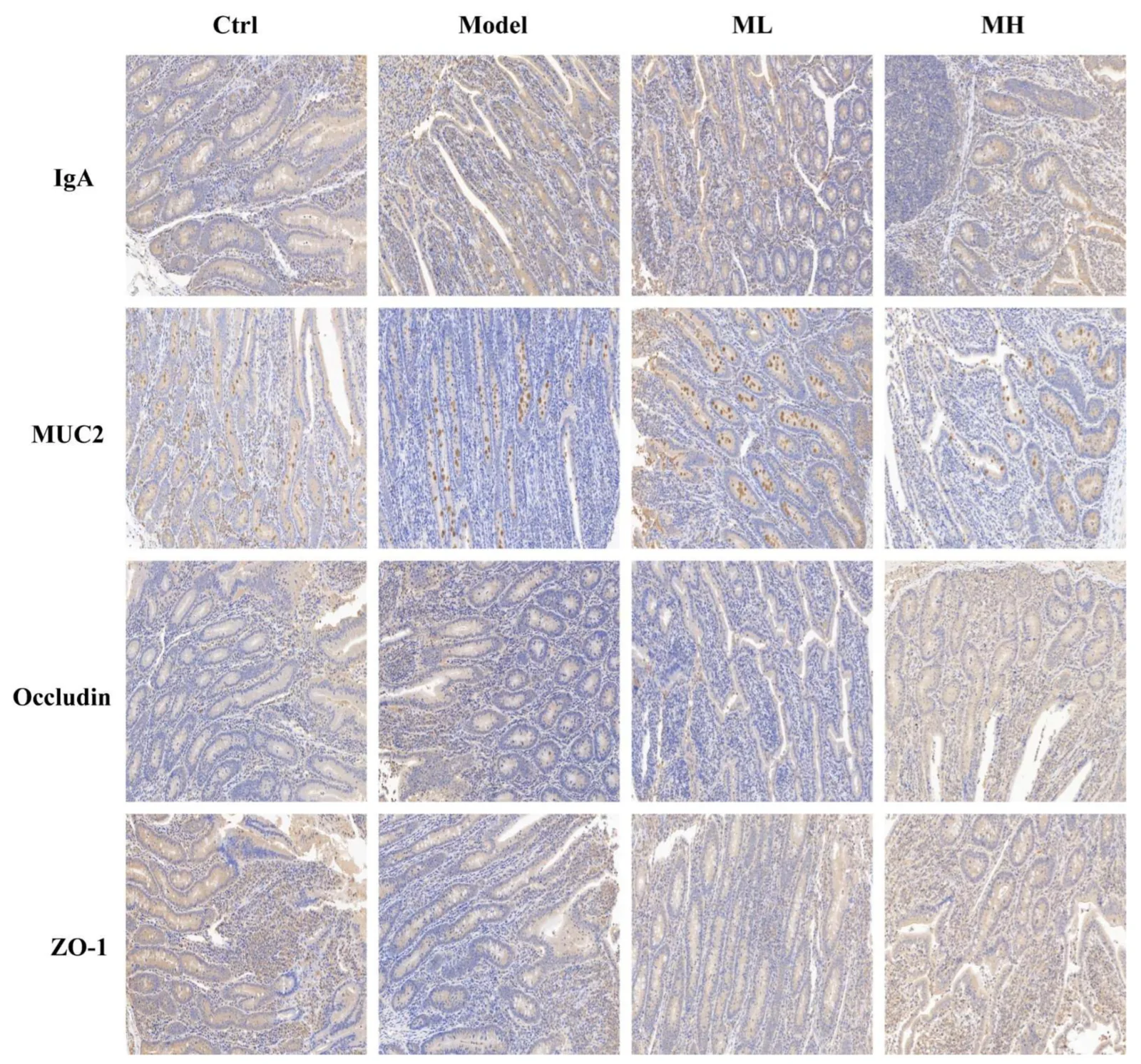
Immunohistochemical analysis of IgA, MUC2, Occludin, and ZO-1 in ileal tissues of Hu sheep. Representative immunohistochemical staining images showing expression of IgA, MUC2, Occludin, and ZO-1 proteins in ileal mucosa from Ctrl, Model, ML, and MH groups. Brown staining indicates positive immunoreactivity. Quantitative results are presented in Table 5. Significant overall effects were marker- and metric-specific (mean intensity: IgA *p* < 0.01; Area%: MUC2 *p* = 0.014), whereas the corresponding Occludin and ZO-1 outcomes were not significant. The caption therefore does not imply generalized enhancement of all barrier-associated proteins.

Immunohistochemistry detected IgA, MUC2, Occludin, and ZO-1 in ileal tissues from all experimental groups (Figure 6).

Representative staining intensity varied among groups, but statistical interpretation should be based on the quantitative measures in Table 5 rather than on image appearance alone.

For mean staining intensity, the overall group effect was significant for IgA (*p* < 0.01) but not for MUC2 (*p* = 0.084), Occludin (*p* = 0.359), or ZO-1 (*p* = 0.123). For positive-area percentage, the overall group effect was significant for MUC2 (*p* = 0.014) but not for IgA (*p* = 0.123), Occludin (*p* = 0.562), or ZO-1 (*p* = 0.170). Therefore, treatment-associated IHC changes were marker- and metric-specific and do not support a generalized enhancement of all barrier-associated proteins.

These immunohistochemical data provide evidence for changes in selected mucosal immune/barrier-associated markers, but they do not directly demonstrate improved epithelial barrier function.

### 3.7. Effects of Dietary A. argyi Inclusion on Rumen Bacterial Community Composition

**Figure 7:**
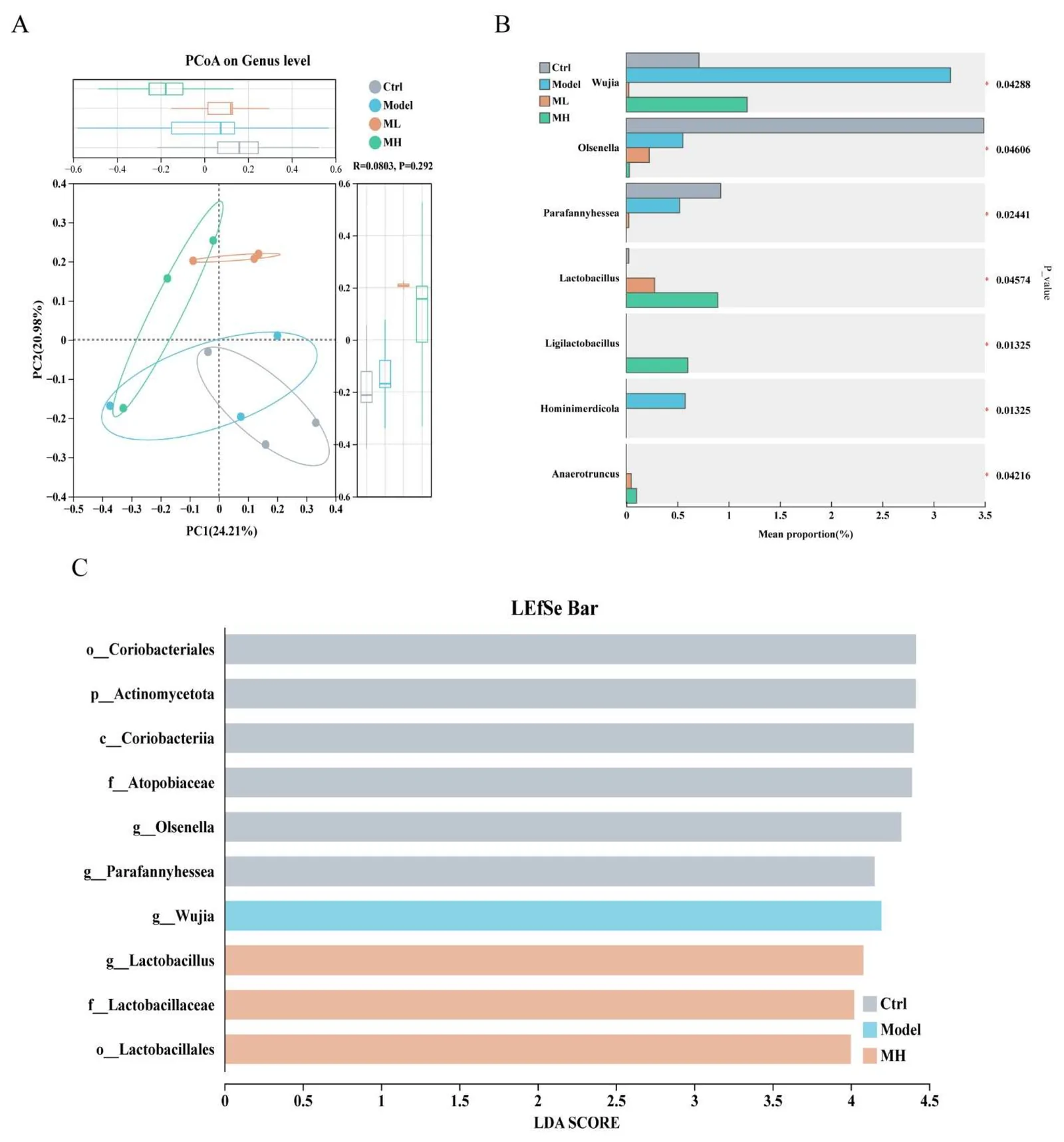
Effects of *Artemisia argyi* inclusion on rumen bacterial community composition in Hu sheep. (**A**) Genus-level PCoA. The overall between-group difference was not significant (R = 0.0803, *p* = 0.292). (**B**) Relative abundance of selected bacterial genera that differed among treatment groups in the analysis shown. (**C**) LEfSe analysis showing treatment-associated differential bacterial taxa; LDA scores are displayed in the panel. Data are presented as mean ± SD (*n* = 3 biological replicates per group): * *p* < 0.05.

Rumen bacterial communities were evaluated by 16S rRNA gene sequencing (Figure 7).

The genus-level PCoA shown in Figure 7A did not demonstrate a statistically significant overall separation among the four groups according to ANOSIM (R = 0.0803, *p* = 0.292), despite visual differences in sample positions. Therefore, the global rumen community should not be described as significantly reshaped on the basis of this PCoA.

The figure supports taxon-specific compositional differences; it does not demonstrate significant global community restructuring or restoration of rumen microbial function.

At the genus level, specific taxa differed among treatment groups (Figure 7B), including Wujia, Olsenella, Parafannyhessea, Lactobacillus, Ligilactobacillus, Hominimerdicola, and Anaerotruncus in the analysis shown.

LEfSe identified treatment-associated differential taxa (Figure 7C). These results support taxon-specific rumen microbial responses, whereas the genus-level PCoA does not support a significant global between-group community difference.

### 3.8. Effects of Dietary A. argyi Inclusion on Colonic Bacterial Community Composition

**Figure 8:**
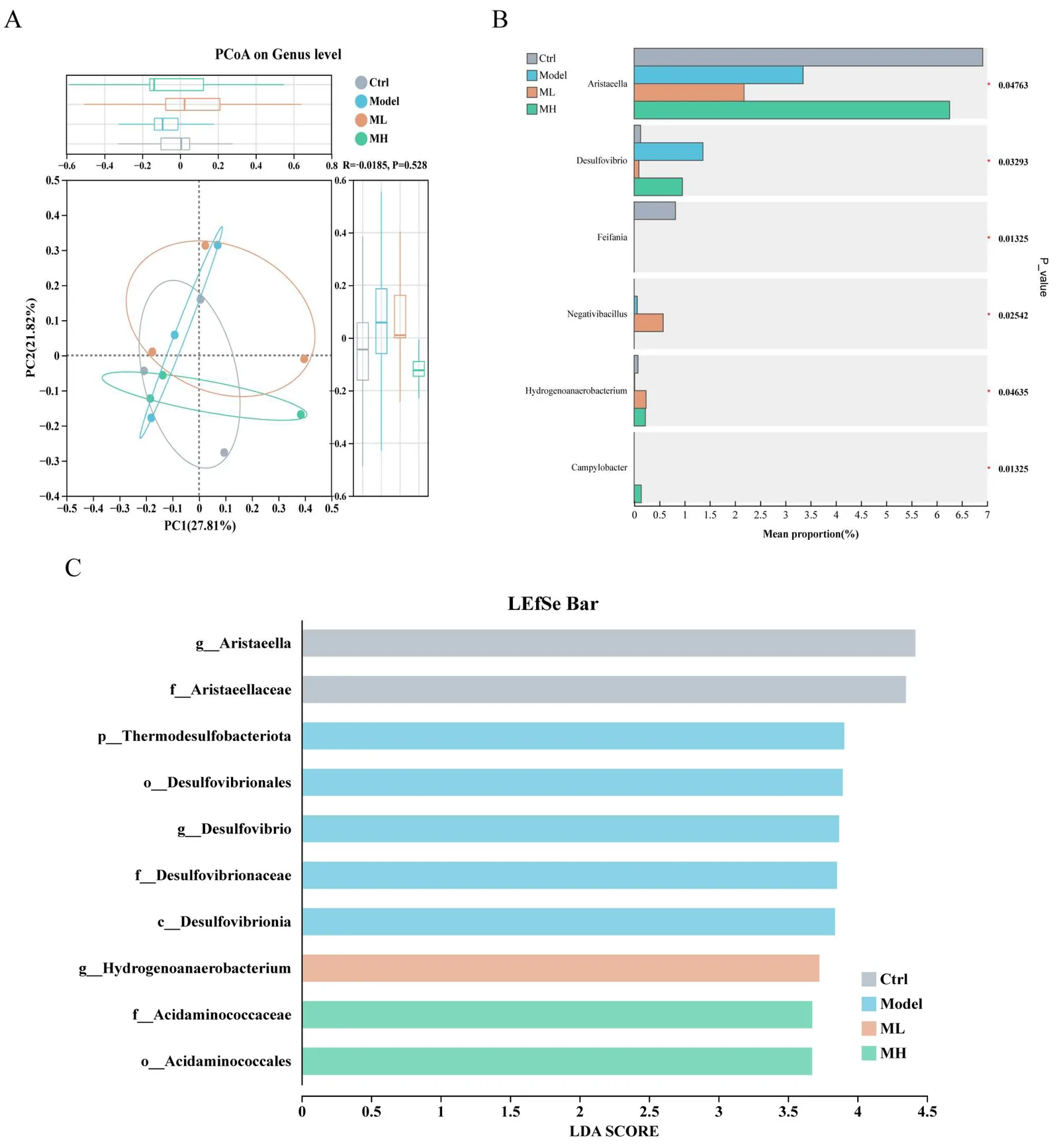
Effects of *Artemisia argyi* inclusion on colonic bacterial community composition in Hu sheep. (**A**) Genus-level PCoA. The overall between-group difference was not significant (R = −0.0185, *p* = 0.528). (**B**) Relative abundance of selected colonic bacterial genera that differed among treatment groups in the analysis shown. (**C**) LEfSe analysis showing treatment-associated differential bacterial taxa; LDA scores are displayed in the panel. The figure supports taxon-specific colonic compositional differences but does not demonstrate significant global community restructuring or microbial functional restoration. Data are presented as mean ± SD (*n* = 3 biological replicates per group): * *p* < 0.05.

The intestinal-content samples used for 16S analysis were collected from the colon; therefore, these data are described as colonic bacterial communities (Figure 8).

The genus-level PCoA shown in Figure 8A did not detect a statistically significant overall separation among the four groups according to ANOSIM (R = −0.0185, *p* = 0.528). Thus, this analysis does not support a statement that gentamicin or *A. argyi* produced a significant global shift in colonic community structure at the genus level.

Specific genera nevertheless differed among groups (Figure 8B), including Aristaeella, Desulfovibrio, Feifania, Negativibacillus, Hydrogenoanaerobacterium, and Campylobacter in the analysis shown.

LEfSe identified treatment-associated differential taxa (Figure 8C). Accordingly, the microbiota findings should be interpreted as taxon-specific compositional differences rather than statistically demonstrated restoration or global restructuring of the colonic bacterial community.

## 4. Discussion

The present study evaluated dietary *Artemisia argyi* inclusion after a gentamicin-associated gastrointestinal disturbance in young Hu sheep using gastrointestinal morphology, epithelial ultrastructure, longitudinal serum immune-related indicators, intestinal inflammatory/barrier-associated markers, and 16S rRNA-based rumen and colonic bacterial profiles. Forty sheep were allocated to four groups (*n* = 10/group) and contributed repeated serum samples, whereas terminal laboratory outcomes were based on three euthanized sheep per group. The findings therefore provide preliminary evidence of treatment-associated responses rather than proof of a unified causal mechanism or population-level efficacy.

Gastrointestinal morphology is closely related to digestive and absorptive capacity in ruminants [20,21,22,23]. In this study, the overall treatment effect on rumen papilla length was significant, but Ctrl and Model were not significantly different according to the reported superscript letters. Small-intestinal responses were segment- and endpoint-specific. For example, jejunal villus length differed from Model in ML but not MH, whereas the ileal V/C ratio differed from Model only in ML. These structural findings do not directly demonstrate improved digestion or absorption because nutrient digestibility, transport, and absorption were not measured. SEM observations were qualitative and are therefore interpreted descriptively rather than as independent statistical evidence.

The response was not consistently dose-dependent. MH showed the largest numerical rumen papilla length, whereas several intestinal endpoints favored ML. With only two inclusion levels and *n* = 3 biological replicates per group for terminal measurements, the study was not designed to define an optimal inclusion level. Additional dose–response studies with larger analytical sample sizes are required.

The rumen and small intestine have distinct physiological roles [1,24,25], which may contribute to the segment-specific morphological responses. However, the present measurements were structural rather than functional. Changes in rumen papillae or intestinal villi should therefore be interpreted as morphological responses associated with *A. argyi* inclusion, not as direct evidence of enhanced fermentation, nutrient absorption, or mucosal protection [26,27].

Maintenance of epithelial integrity is essential for gastrointestinal homeostasis [28,29,30,31,32,33]. In the present study, qPCR responses were gene- and segment-specific: Claudin-1 expression showed little separation in the duodenum and was lower after *A. argyi* inclusion than in the control model in the jejunum and ileum. IHC effects were also marker- and metric-specific. Mean IgA staining intensity and MUC2-positive area showed significant overall group effects, whereas the corresponding Occludin and ZO-1 outcomes were not significant. These findings support changes in selected barrier-associated markers but do not establish restoration of intestinal barrier function; direct permeability and functional barrier assays would be required.

Immune and inflammatory responses are closely linked to gastrointestinal disturbance [34,35]. Serum cytokines and immunoglobulins varied over time, and intestinal inflammatory gene expression differed by gene and segment. The serum data do not support a uniform anti-inflammatory or immunostimulatory effect across all markers and time points, and the qPCR data do not support a uniform response across all intestinal segments. Longitudinal serum interpretation should therefore be based on the repeated-measures group, time, and group × time effects rather than on isolated numerical differences.

The combination of serum immune indicators and ileal IHC provides information on systemic and local mucosal-associated responses [36]. However, serum immunoglobulin concentrations do not directly represent secretory immune responses in the intestinal lumen [37], and the IHC data showed significant effects only for selected marker/metric combinations. Future studies should include secretory IgA, local cytokine protein measurements, mucosal immune-cell phenotyping, and functional barrier measurements.

The gastrointestinal microbiota contributes to digestive and physiological homeostasis in ruminants [1,38]. Genus-level PCoA followed by ANOSIM did not show significant global between-group separation for either rumen (R = 0.0803, *p* = 0.292) or colonic communities (R = −0.0185, *p* = 0.528). Taxon-level comparisons and LEfSe nevertheless identified treatment-associated taxa. The bacterial-community results therefore support taxon-specific compositional differences rather than statistically demonstrated global community restoration. Moreover, 16S rRNA sequencing does not establish microbial metabolic activity, pathway function, or causality.

Several detected taxa may provide hypotheses for future work. Lactobacillus and Ligilactobacillus varied in the rumen analysis, whereas the colonic analysis included Desulfovibrio and other genera. Although these taxa have been linked to carbohydrate fermentation, mucosal microecology, or sulfur metabolism [39,40,41,42], such associations should be interpreted cautiously [43,44]. The observed taxa are best regarded as treatment-associated bacterial signatures without complementary metabolomic, metagenomic, or culture-based validation.

A strength of this study is the concurrent assessment of morphological, immune-related, barrier-associated, and bacterial-community endpoints within the same experimental framework. However, concordant changes across domains do not demonstrate causal links among them. The data cannot determine whether bacterial differences preceded host responses, whether host changes altered the microbial environment, or whether both occurred independently after *A. argyi* inclusion. Mechanistic language is therefore limited to associations supported by the measured endpoints.

Several limitations should be acknowledged. First, although 10 sheep were allocated to each treatment group and all sheep contributed repeated serum samples, only three sheep per group were euthanized for terminal laboratory sampling; this small terminal analytical sample size limits precision and statistical power and increases sensitivity to between-animal variation. Second, the commercial feed information currently available from the manufacturer did not disclose the ingredient formulation or ingredient-level proportions. Third, some methodological metadata could not be verified from the available records, including the gentamicin product manufacturer, unambiguous IgM and TNF-α ELISA kit identifiers, and the SEM make/model. Fourth, 16S rRNA sequencing characterized bacterial composition but not microbial metabolites or functional pathways, and the genus-level PCoA/ANOSIM analyses were not significant globally. Fifth, intestinal permeability was not measured directly, and the bioactive constituents of the whole *A. argyi* material were not quantified. Finally, growth performance, feed intake, nutrient digestibility, and feed efficiency were not evaluated. These limitations should be considered when interpreting the findings.

Future studies should use a priori sample-size calculations based on a prespecified primary endpoint, increase terminal analytical replication, retain complete treatment and assay metadata, and integrate direct intestinal permeability measurements, production-performance outcomes, and functional microbial analyses. The present results support further investigation of *A. argyi* as a feed additive after antibiotic-associated gastrointestinal disturbance rather than a definitive claim of gastrointestinal recovery.

## 5. Conclusions

Dietary inclusion of *Artemisia argyi* after gentamicin exposure was associated with selected gastrointestinal responses in young Hu sheep. *A. argyi* inclusion altered specific rumen and intestinal morphological indices, longitudinal serum immune-related profiles, intestinal inflammatory/barrier-associated markers, and the relative abundance of selected rumen and colonic bacterial taxa. Responses varied among gastrointestinal segments and inclusion levels. The genus-level PCoA/ANOSIM analyses did not demonstrate significant global separation of rumen or colonic bacterial communities. Serum outcomes were based on *n* = 10 sheep per group, whereas terminal tissue and content outcomes were based on *n* = 3 biological replicates per group. These findings support further evaluation of *A. argyi* as a natural feed additive but do not establish uniform improvement of gastrointestinal function or microbial restoration.

## Figures and Tables

**Table 1:** Manufacturer-Declared Nutrient Specifications of the Commercial Basal Diet for Early-Fattening Hu Sheep.

Items	Label Specification (%)
Crude protein	≥13.00
Crude fiber	≤25.00
Crude ash	≤15.00
Calcium (Ca)	0.50–2.50
Moisture	≤10.00
Total phosphorus (P)	≥0.20
Sulfur-containing amino acids	≥0.30

Note: Values were obtained from the product label and manufacturer inspection report and are reported on an air-dried basis. The symbols ≥ and ≤ indicate guaranteed minimum and maximum values, respectively. The available product documentation did not provide the ingredient list or ingredient-level proportions for product 198; therefore, these data are not reported or estimated.

**Table 2:** Sequences of primers used in the gene expression analysis.

Primer	Sequence (5′-3′)
IFN-γ	F: TTCTTGAACGGCAGCTCTGA R: CTCCGGCCTCGAAAGAGATT
IL-6	F: CCTGTCCACTGGGCACATAA R: AAGGACGTTCAAGCCGCATA
IL-17	F: ATCTACAGTGAACTGGAAGGAG R: CGAAGGACCAGGATCTCTTGCT
TNF-α	F: ACTCGTATGCCAATGCCCTC R: CAAGGGCTCTTGATGGCAGA
Claudin-1	F: GGCTTCATCCTGGCGTTTCT R: TTGCTTGCAGAGTGCTGTTC
β-actin	F: CCTCACTGCTTCCTTCTCTCTC R: CCTAGAAGCATTTGCGGTGG

**Table 3:** Effects of *Artemisia argyi* inclusion on the Morphological Characteristics of Rumen Mucosa in Hu Sheep.

Group	Papilla Length (mm)	Papilla Width (mm)	Mucosal Thickness (mm)
Ctrl	0.70 ± 0.23 ^ab^	0.05 ± 0.37	0.49 ± 0.14
Model	0.48 ± 0.26 ^a^	0.09 ± 0.3	0.51 ± 0.24
ML	0.96 ± 0.45 ^bc^	0.06 ± 0.39	0.54 ± 0.18
MH	1.16 ± 0.68 ^c^	0.06 ± 0.35	0.49 ± 0.18
*p*-value	<0.001	>0.05	>0.05

Note: Data are presented as mean ± SEM. Different superscript letters indicate significant differences among groups (*p* < 0.05).

**Table 4:** Effect of *Artemisia argyi* inclusion on the Histological Structure of the Small Intestine in Hu Sheep.

Intestinal Segment	Group	Villus Length V (μm)	Crypt Depth C (μm)	V/C
Duodenum	Ctrl	429.33 ± 87.90 ^b^	279.07 ± 39.64 ^b^	1.58 ± 0.42
	Model	292.53 ± 94.24 ^a^	215.8 ± 66.47 ^a^	1.97 ± 2.46
	ML	423.73 ± 72.2 ^b^	240.8 ± 48.8 ^ab^	1.8 ± 0.4
	MH	480.67 ± 75.54 ^b^	225.2 ± 58.62 ^a^	2.26 ± 0.67
	*p*-value	<0.001	0.013	0.545
Jejunum	Ctrl	530.8 ± 54.83 ^b^	419.6 ± 84.32 ^a^	1.3 ± 0.23
	Model	427.2 ± 57.53 ^a^	559.87 ± 113.95 ^b^	0.79 ± 0.21
	ML	688.8 ± 129.22 ^c^	410.13 ± 79.23 ^a^	1.73 ± 0.43
	MH	468.6 ± 60.89 ^a^	434.67 ± 51.64 ^a^	1.09 ± 0.2
	*p*-value	<0.001	<0.001	<0.001
Ileum	Ctrl	383.33 ± 43.45 ^a^	288.07 ± 49.48 ^a^	1.37 ± 0.27 ^a^
	Model	412.13 ± 82.22 ^ab^	354.07 ± 100.95 ^b^	1.36 ± 0.88 ^a^
	ML	766.4 ± 128.66 ^b^	337.47 ± 43.5 ^ab^	2.32 ± 0.53 ^b^
	MH	469.27 ± 70.44 ^c^	311.13 ± 47.2 ^ab^	1.54 ± 0.29 ^a^
	*p*-value	<0.001	<0.05	<0.001

Note: Data are presented as mean ± SEM. Different superscript letters indicate significant differences among groups (*p* < 0.05).

**Table 5:** Effects of *Artemisia argyi* inclusion on Immunohistochemical Expression of IgA, MUC2, Occludin and ZO-1 in the Ileum of Hu Sheep.

	Group	IgA	MUC2	Occludin	ZO-1
Mean	Ctrl	135.12 ± 4.94	120.86 ± 5.53	121.25 ± 3.1	144.7 ± 11.34
	Model	96.55 ± 15.48	121.35 ± 11.37	93.96 ± 10.25	113.86 ± 13.79
	ML	173.11 ± 8.37	161.72 ± 9.75	170.86 ± 7.62	166.54 ± 4.71
	MH	167.2 ± 9.75	177.45 ± 2.95	172.08 ± 3.17	166.55 ± 1.96
	*p*-value	<0.01	0.084	0.359	0.123
Area%	Ctrl	1.89 ± 0.28	1.35 ± 0.61 ^a^	1.32 ± 0.13	2.43 ± 0.89
	Model	1.09 ± 0.27	2.46 ± 0.46 ^c^	2.13 ± 0.64	1.39 ± 0.13
	ML	8.54 ± 0.54	6.86 ± 1.94 ^bc^	6.18 ± 2.84	6.98 ± 2.05
	MH	13.59 ± 3.63	10.17 ± 1.46 ^ab^	7.35 ± 1.59	12.99 ± 5.27
	*p*-value	0.123	0.014	0.562	0.17

Note: Data are presented as mean ± SEM. Different superscript letters indicate significant differ-ences among groups (*p* < 0.05).

## Data Availability

The original contributions presented in this study are included in the article. Further inquiries can be directed to the corresponding authors.
